# Polylactide (PLA) Filaments a Biobased Solution for Additive Manufacturing: Correlating Rheology and Thermomechanical Properties with Printing Quality

**DOI:** 10.3390/ma11071191

**Published:** 2018-07-11

**Authors:** Gianluca Cicala, Davide Giordano, Claudio Tosto, Giovanni Filippone, Antonino Recca, Ignazio Blanco

**Affiliations:** 1 Dipartimento DICAR (Department of Civil Engineering and Architecture), Università di Catania, Viale Andrea Doria 6, 95125 Catania, Italy; gcicala@unict.it (G.C.); Iblaprotolab@gmail.com (D.G.); claudio089@hotmail.it (C.T.); arecca@dii.unict.it (A.R.); 2 UdR-Catania Consorzio INSTM, Viale Andrea Doria 6, 95125 Catania, Italy; 3 Dipartimento di Ingegneria Chimica, dei Materiali e Della Produzione Industriale, Università degli Studi di Napoli Federico II, Piazzale Tecchio 80, 80125 Napoli, Italy; gfilippo@unina.it; 4 UdR-Napoli Consorzio INSTM, Piazzale Tecchio 80, 80125 Napoli, Italy

**Keywords:** additive manufacturing, fused deposition modeling, rheology, mechanical properties, PLA, 3D printing, thermal properties

## Abstract

Three commercial filaments for Fused Deposition Modeling (FDM) were selected to study the influence of polymer formulation on the printing quality and mechanical properties of FDM specimens. The three filaments were all based on polylactic acid (PLA) as the matrix, and they are sold as PLA filaments. The printing quality was tested by printing one complex shape with overhang features. The marked shear thinning behavior for two filaments was observed by rheology. The filaments were also studied by scanning electron microscopy and thermogravimetric analysis (TGA) to unveil their composition. The filaments with the best printing quality showed the presence of mineral fillers, which explained the melt behavior observed by rheology. The tensile testing confirmed that the filled PLA was the best-performing filament both in terms of printing quality and thermomechanical performance, with a *p*-value = 0.106 for the tensile modulus, and a *p*-value = 0.615 for the ultimate tensile strength.

## 1. Introduction

Additive manufacturing (AM) has been increasingly accepted in industry [1,2,3]. Worldwide market for AM products and services is estimated to grow to over $5 billion by 2020 [4]. The filament-based technology referred as the Fused Deposition Modeling (FDM) is one the also widely used, mostly for functional parts [5,6,7,8,9,10,11]. Knowledge of the impact of material properties and the mechanical properties of printed parts is of paramount importance to further expand the use of FDM.

In Fused Deposition Modeling, thermoplastic filaments for both model and support are fed into the heated extrusion print head, enabling three-dimensional (3D) dispensing of the resulting polymer melts on a platform, which is lowered step-by-step once each object’s layer is completed. Layer by layer, the object is then printed. The FDM process is controlled by many parameters, which range from material filament type to machine setting such as: nozzle diameter and temperature, printing speed, feed rate, bed temperature, raster angle and width, etc. Detailed studies are reported in the literature on the influence of the printing settings on mechanical properties [12,13,14,15,16]. Zaldivar et al. [13], for example, showed recently that Ultem 9085-printed specimen mechanical properties are significantly affected by build orientation. The strength utilization in terms of FDM/injection-molded performance can vary from 85.8% for edge-printed specimens to 46.5% for samples printed upright. Similar results were evidenced by Cicala et al. [17], thus suggesting that the final properties of the FDM-printed part are highly anisotropic. This anisotropy has a relevant effect on the final performances of a printed part. In addition, the lower performances compared with injection-molded specimens are also the result of the intrinsic voids in FDM-printed parts between the fused filaments due to poor bonding among wires [15,16]. Motaparti et al. [14] showed that the strength of a thermoplastic interface within the FDM part is directly proportional to the intermolecular diffusion across the interface between the fused filaments. The relevance of the bond quality between adjacent filaments depends on the printing parameters, as well as on the melt viscosity of the polymer used for the filaments. However, to the best of our knowledge, the impact of rheology on the FDM printing process has been only investigated in the literature by few studies, such as those from McIlroy et al. [18,19], Murphy and Collins [20], and Cicala et al. [21]. Therefore, a major point deserving of a more detailed analysis is the study of the rheological behavior of polymer melts during FDM processing to rationalize the effect of melt behavior on printing quality.

In the present paper, three different commercial polylactide (PLA) filaments were selected and printed with a commercial FDM printer, selecting a challenging 3D geometry with overhang printing. The filaments were thoroughly characterized by rheological, microscopic, and thermal techniques to explain the differences in the printing and tensile properties observed.

## 2. Experimental

### 2.1. Materials and Methods

#### 2.1.1. Materials

Three industrial PLA-based filaments for FDM printing were purchased from different commercial sources. The three materials had different colors: white for the PLA Ultra by Roboze (Bari, Italy); black for the PLA Layer by Stampa3DSud (Salerno, Italy), and green for the PLA by Stampa3dSud (Salerno, Italy).

#### 2.1.2. Specimens Manufacturing

**Figure 1:**
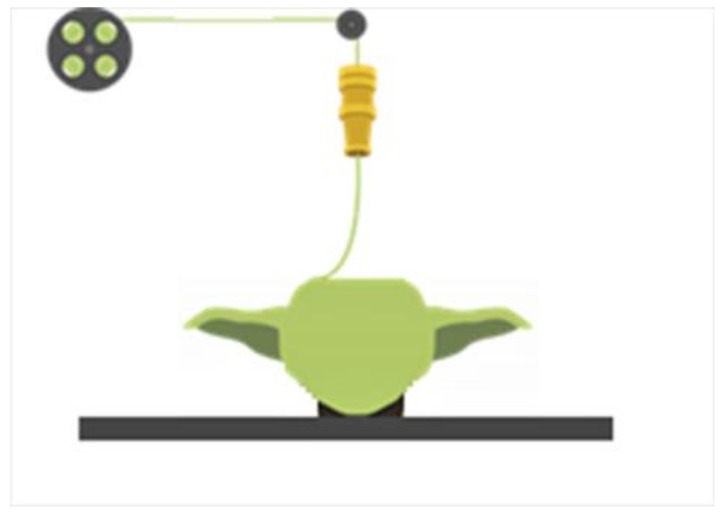
Cartoon showing the Yoda sample while printing.

The printing efficiency of the three filaments was tested by printing a complex geometry (i.e., Yoda face) without the use of a support material (Figure 1). These test trials are routinely used to assess the so-called overhang printing behavior. The FDM machine Zortrax M200 (Zortrax, Olsztyn, Poland) was used for all of the printing trials. The conditions for printing the Yoda specimens and the specimens for tensile tests are summarized in Table 1. Tensile specimens were oriented flatwise in the XY plane.

### 2.2. Characterization

#### 2.2.1. Mechanical Testing

Tensile specimens were printed accordingly to ASTM D638. The tensile properties of the printed materials were investigated by testing five samples of each kind of material under study. The tensile properties of printed specimens were measured by using an Instron 5985 universal testing machine that was equipped with a load cell of 10 kN. Specimens were tested at a constant rate of 2 mm/min, while compliance correction was used. System control and data analysis were performed using Blue Hill 3.61 software (Instron, Norwood, MA, USA).

The differences in mechanical results were statistically analyzed by one-way analysis of variance (ANOVA) using Minitab 17 software (GMSL S.r.l. Milano, Italy). To identify which groups were significantly different from other groups, means comparison was done using Fisher’s test with a 95% confidence level (see Supplementary Materials).

#### 2.2.2. Scanning Electron Microscopy (SEM)

Cryofractured surfaces of the as received filaments were analyzed with an EVO Scanning Electron Microscope (EVO-SEM, Zeiss, Cambridge, UK). All of the samples were gold sputtered up to a thickness of 20 nm by means of an Emitech K-550 sputter coater (Ashford Kent, UK). An accelerating voltage of 15 kV was used to collect the micrographs.

#### 2.2.3. Rheological Analysis

Rheological tests were performed in a wide range of shear rate ($\dot{\gamma }$) using both rotational and capillary rheometer. The low-shear rate behavior and the linear viscoelasticity of the materials were investigated using a stress-controlled rheometer (ARG2 by TA Instruments, New Castle, DE, USA) in parallel-plate configuration (plate diameter 25 mm). The pellets obtained by the filaments were loaded in the cavity constituted by the lower plate and a containment ring. The upper plate was then lowered for compacting the pellets, and the containment ring was finally removed. The previous procedure, which was carried out at T = 210 °C in nitrogen atmosphere, led to disk samples with thicknesses of about 1 mm that were ready for rheological tests. The shear viscosity (*η*) was estimated by performing steady-state flow tests. The elastic (*G*′) and viscous (*G*″) shear moduli were collected by means of frequency scan experiments at strain amplitudes low enough to be in the linear regime. The latter was evaluated through preliminary strain amplitude tests. All of the experiments were performed at T = 210 °C in nitrogen atmosphere. The high-shear rate behavior was investigated using a capillary rheometer (CEAST SR20 by Instron, Norwood, MA, USA). Tests were carried out at 210 °C using capillaries with 1-mm diameter and different lengths. Both Bagley and Mooney–Rabinowitsch corrections were performed.

#### 2.2.4. Thermal Analysis

A Shimadzu (Kyoto, Japan) DTG-60 simultaneous DTA-TG apparatus was used for both thermogravimetric analysis (TGA) and differential thermal analysis (DTA). The calibrations of temperature, heat flow, and weight were performed following the procedure suggested by the manufacturer and reported elsewhere [22,23], using as standard materials: indium (NIST SRM 2232), tin (NIST SRM 2220), and zinc (NIST SRM 2221a) for temperature; indium (NIST SRM 2232) for heat flow; and a set of exactly weighed samples supplied by Shimadzu for weight. TGA scans were performed from 25 to 600 °C, at the heating rate of 10 °C∙min^−1^, under flowing nitrogen (0.02 L∙min^−1^). Samples of about 10 × 10^−3^ g, which were placed in a 40-μL alumina open pan, were degraded as a function of temperature, and their mass loss was recorded by the DTG-60 apparatus. The experimental data were then used to plot the percentage of the undegraded sample W/W_o_% as a function of temperature, where W_o_ and W were the weights of the sample at the starting point and during scanning. In order to correct the error in the mass determination due to the reduction of the buoyancy force with increasing temperature [24], TGA measurements were performed by adopting the blank method. For DTA analysis, the heat flow of sample was monitored and recorded by the same DTG-60 apparatus to evaluate the enthalpy and temperature of the observed phase transitions. All of the considered values were averaged from three runs, with the maximum difference between the average and the experimental values being within ±1 °C.

#### 2.2.5. IR Spectroscopy

The solid residues obtained from the TGA measurements at 600 °C were analyzed with a Perkin Elmer Spectrum 100 spectrometer (Perkin Elmer, Waltham, MA, USA), and the Fourier transform infrared (FTIR) spectra were traced. Spectra were carried out directly on samples at r.t. without any preliminary treatment, using an universal ATR sampling accessory, from 4000 to 600 cm^−1^, with a resolution of 4.0 cm^−1^.

## 3. Results and Discussion

### 3.1. 3D Printing of PLA Filaments

**Figure 2:**
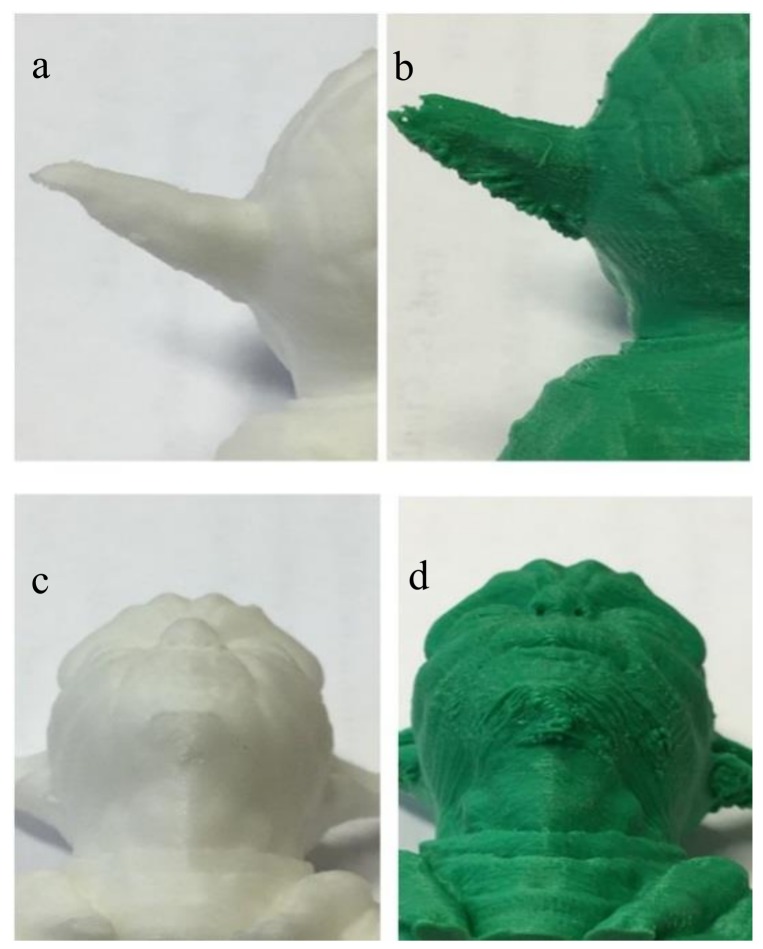
Some examples of the overhang printing defects due to uncontrolled flow of the polymer: the white Yoda shows no defect (**a,c**); the green Yoda showed printing defects in the critical area such as ears and the chin (**b,d**).

The three PLA filaments were used to print the Yoda sample without the use of any support and by using always the same g-code. The Yoda shape has some critical printing issues with some selected parts, such as the ear and the chin, because of the absence of support materials (Figure 1). Such printing is usually referred to technically as overhang printing. These parts are critical, because the flow of the melted polymer with no control of sag properties can result in printing defects (Figure 2). These defects resemble those that are commonly reported as blobs and zits. In our Yoda-printed samples, printing defects occurred only with the green PLA, whilst by using both the white and the black PLA, no visible defect was observed. The three PLAs were further analyzed by rheology, scanning electron microscopy, and thermal analysis to unveil the underlying structure–property correlations.

### 3.2. Rheological Properties of PLA Filaments

**Figure 3:**
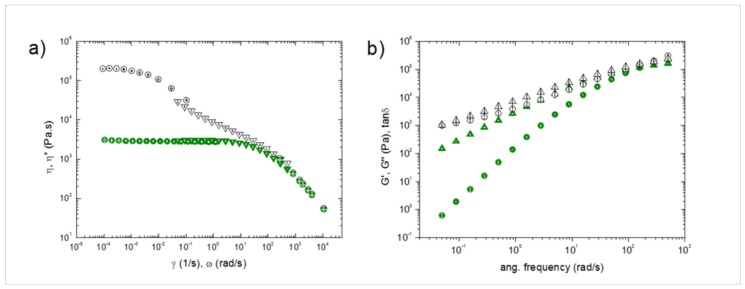
(**a**) Shear (circles) and complex (triangles) viscosity for white (open symbols) and green (full symbols) polylactide (PLA). (**b**) Elastic (circles) and viscous (triangles) modulus for white (open symbols) and green (full symbols) PLA.

An approximated estimate of the shear rate experienced by the polymer when passing through the nozzle leads to $\dot{\gamma }$ of an order of 10^4^ 1/s (see Supplementary Materials). Capillary rheometer experiments were performed to access such a high shear rate regime. The results are shown in Figure 3a. Since the black and the white PLA exhibited very similar rheological behavior, the data for the black sample are not shown for the sake of clarity. The three polymers exhibited comparable values of the viscosity for $\dot{\gamma }$ > 10^3^ 1/s, sharing the same power-law behavior, $\eta \sim {\dot{\gamma }}^{-0.78}$. On the other hand, noticeable differences emerged when lower shear rates were considered. In this regime, the data were collected via rotational rheometry. The white (and black) sample exhibited $\dot{\gamma }$-independent viscosity at $\dot{\gamma }$ < 10^−3^ 1/s ($\eta_{0}≅7\times 10^{4}$ Pa.s), whilst η rapidly decreased above this threshold. Differently, the green PLA exhibited Newtonian behavior up to $\dot{\gamma }$~10^0^ 1/s, with a viscosity that was two orders of magnitude lower than that of the other two samples. This difference is likely correlated to the different processability of the materials in the course of FDM.

To deepen the rheological analysis, small amplitude oscillatory shear tests were performed. The results are reported in terms of both complex viscosity (η*) (Figure 3a) and linear viscoelastic moduli (Figure 3b). The Cox–Merz rule, which typically applies for pure polymers, predicts that $\eta^{*}\left(\omega \right)=\eta \left(\dot{\gamma }\right)$ for $\omega =\dot{\gamma }$ [25]. A good overlapping between *η* and η* was noticed for the green PLA, whilst the Cox–Merz rule failed for the white and the black PLA. Looking at the viscoelastic moduli, the typical Maxwellian behavior that is expected for pure polymers (*G*’~*ω*^2^ and *G*″~*ω*^1^ at low frequency) was observed for the green PLA, which behaved similar to a viscous liquid with negligible elasticity. In contrast, the white and the black samples exhibited a marked elasticity and a much weaker ω-dependence of the moduli. This is indicative of a considerable amount of additives, which confers a marked solid-like behavior to the melt.

To summarize, more than differences in the rheological behavior during the passage through the nozzle, the different processability of the three samples with FDM technique appeared to be correlated to the rheological properties outside the printing apparatus, when the filament settled layer-by-layer experiencing slow deformation rates. In this regime, remarkable differences were observed for the different samples. In particular, the low viscosity and negligible elasticity of the green PLA resulted in a much higher propensity to flow/drip soon after the filament deposition compared with the other samples, which were much more structured fluids, which was probably due to the presence of additives in the form of solid particulate. This explains the poor printing quality of the green PLA, with blobs and zits observed in the overhang printed regions; in contrast, despite the comparable viscosity shared by all of the samples when passing through the nozzle, the black and the white polymers have kept their shape while depositing the filament, and this limited the imperfections and defects in the artifact.

### 3.3. Morphological Analysis of PLA Filaments

**Figure 4:**
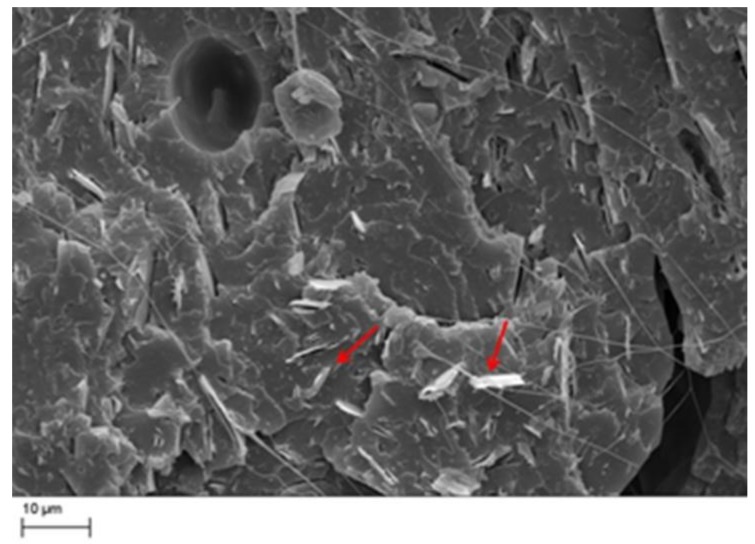
Scanning electron microscope of the white PLA filament. Red arrows mark some of the inorganic fillers (magnification 2.5kX).

Scanning electron microscope on cryofractured filaments was performed. The results of the SEM analysis of the white PLA is reported in Figure 4. The picture clearly shows the presence of some white fillers marked by red arrows.

### 3.4. Thermal Analysis of the PLA Filaments

**Figure 5:**
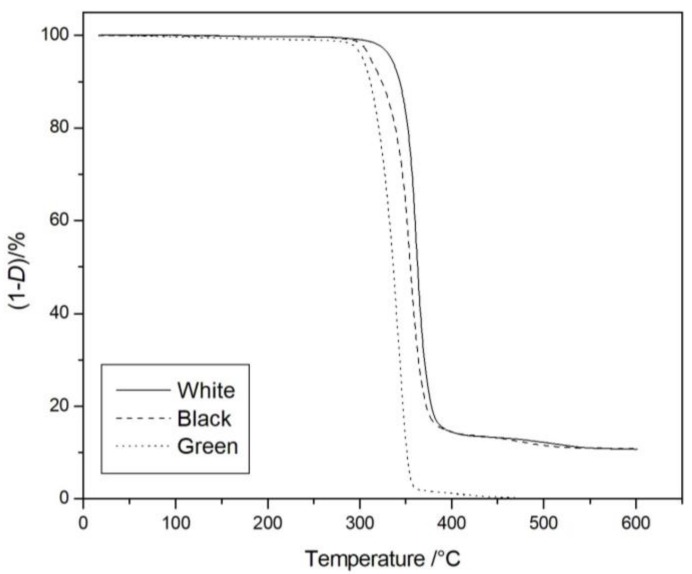
Thermogravimetric analysis (TGA) curves of the three PLA commercial samples.

The thermal behavior of our compounds was studied by TGA and DTA experiments in the temperature range of 35 °C to 600 °C, in flowing nitrogen. The thermogravimetric curves (Figure 5) evidenced a single-stage degradation mechanism for all three samples. Whilst the green sample was completely degraded at about 450 °C, both the white and black samples showed a stable residue of the same magnitude up to 600 °C, which confirmed the presence of mineral fillers in these PLA samples, which is in agreement with literature evidence that showed the complete degradation of neat PLA at those temperatures [26,27,28], and with the findings of SEM analysis.

**Figure 6:**
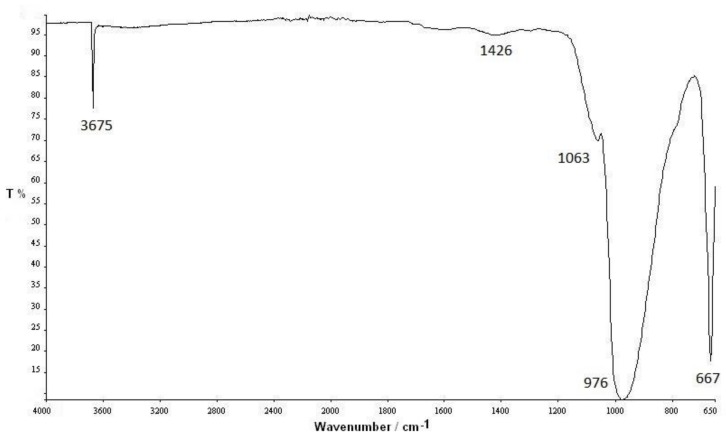
Fourier transform infrared (FTIR) spectra of the solid residue at 600 °C from the degradation of the white sample.

Moreover, from the analysis of the TGA curves, it is evident that the beginning of the degradation of the white sample clearly shifted toward higher temperatures compared wtih the black and green samples, respectively. This behavior was confirmed quantitatively by the calculation of the temperatures at 5% mass loss (*T*_5%_), which are a measure of their thermal stability. *T*_5%_ values were reported in Table 2 together with the residue percentage at 600 °C, showing an increment for the white sample of about 30 °C and 25 °C in respect to the black and green samples respectively. The residue content was stable up to 600 °C, confirming its nature of mineral filler, which was clearly observed by the microscopic analysis of Figure 4. The most probable filler used in such filaments was talc, considering the shape observed from SEM analysis (Figure 4). In order to confirm this hypothesis, FTIR analyses on the solid residue obtained at 600 °C were thus carried out, and the spectrum for the white sample is reported, as shown in Figure 6.

The characteristic peaks at 3670, 970, and 665 cm^−1^ are shown, thus confirming the presence of talc [29].

**Figure 7:**
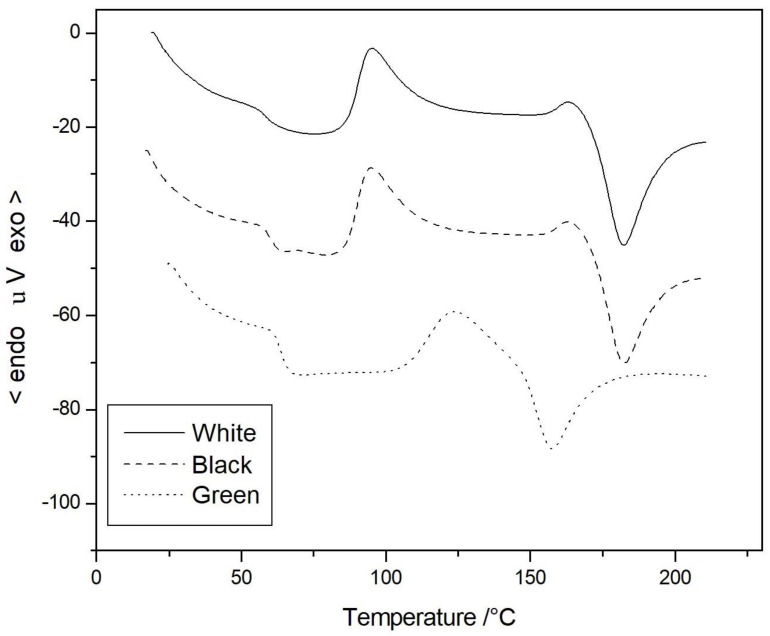
Differential thermal analysis (DTA) curves of the three PLA commercial samples.

The DTA analyses confirmed the different thermal behavior among the investigated compounds. White and black samples showed two almost superimposable DTA traces (Figure 7), with the same values of glass transition (*T*_g_), crystallization (*T*_c_), and melting temperature (*T*_m_) as reported in Table 2. The melting and crystallization temperatures observed for the white and the black samples fit with the values reported in the literature for similar microcomposites of PLA filled with talc or even wood flour [30,31], thus ensuring a good processability. By contrast, the DTA curve of the green sample was completely different, showing an increase in the *T*_g_ value, but a dramatic decrease in the melting behavior, which was about 20 °C less than the two other samples. This difference can be due to either a different molecular weight for the green PLA, or the presence of some copolymers in this filament.

### 3.5. Mechanical Properties of PLA-Printed Specimens 

**Figure 8:**
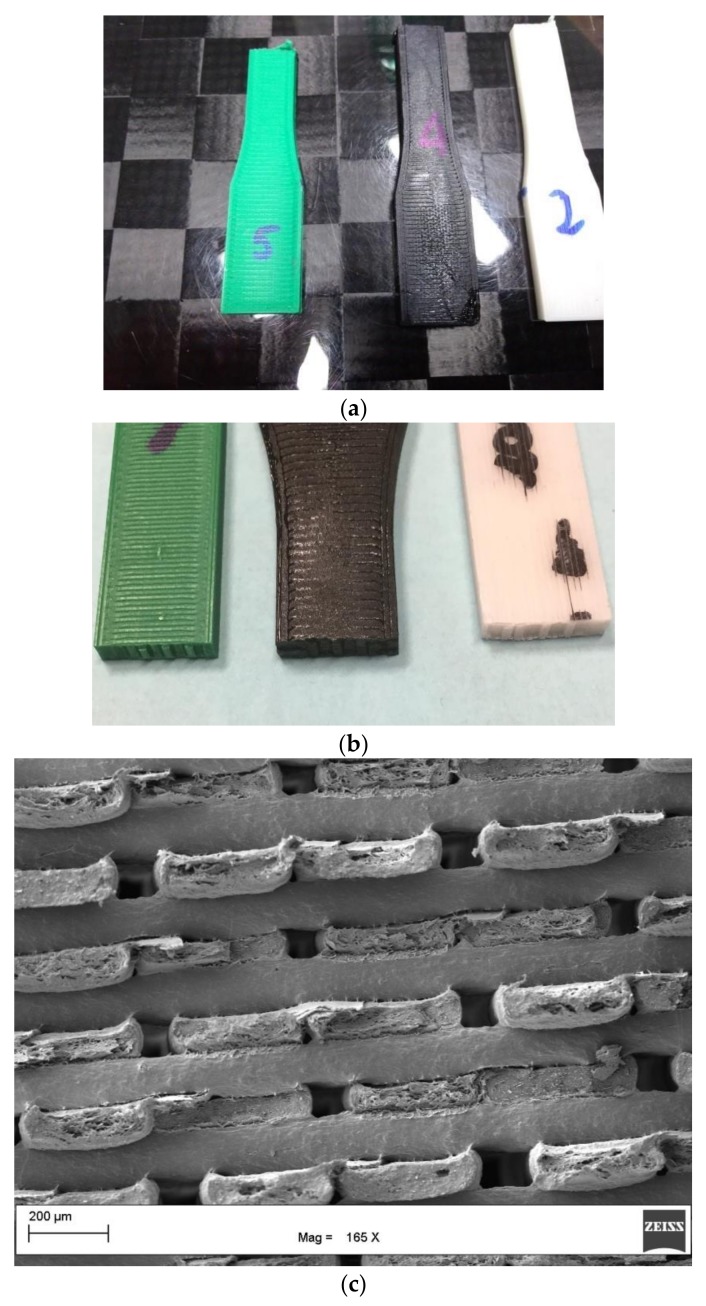
Printed tensile specimens: (**a**) surface quality; (**b**) tensile fractured surface; (**c**) SEM analysis of the tensile fractured white specimen.

The printed specimens that showed a high surface quality (Figure 8a) were tested in tension mode to verify the mechanical performances, and the data obtained for all three samples are reported in Table 3.

The samples displayed brittle failure mode (Figure 8b) with limited deformation at break, confirming the results summarized in Table 3. SEM analysis of the tensile fractured specimen (Figure 8c) confirmed the absence of extensive yielding, as observed optically (Figure 8b).

**Figure 9:**
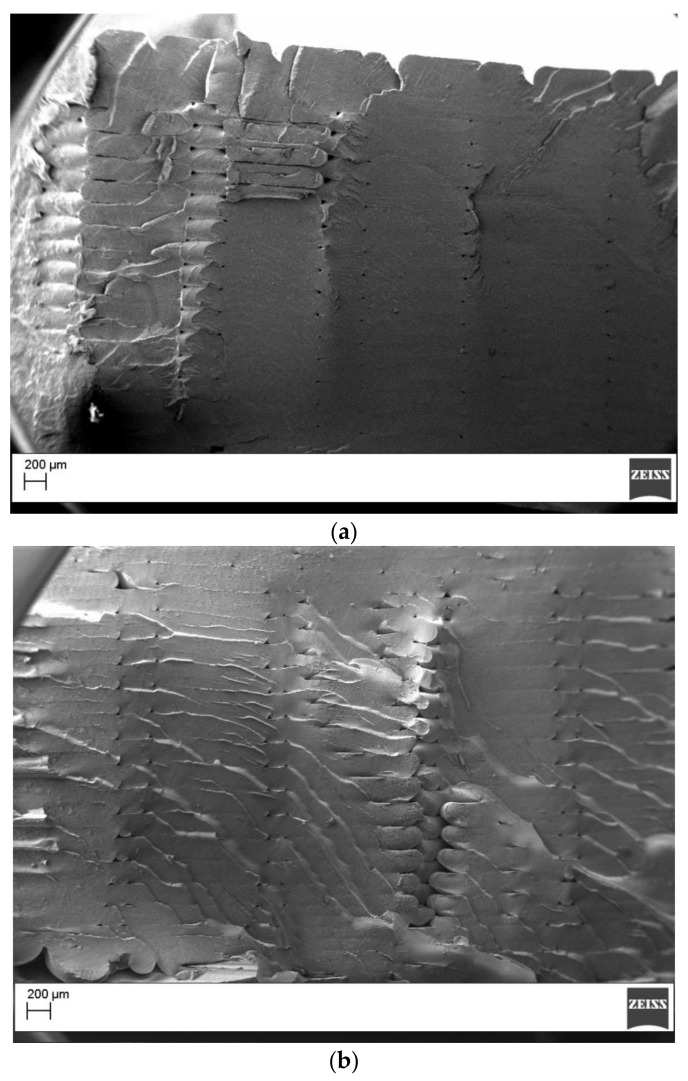
Cryofactured surface for the tensile specimens: (**a**) green; (**b**) black (magnification 50X).

The tensile specimens were fractured cryogenically, and the SEM analysis, as reported in Figure 9, confirmed the good quality printing with the beads displaying an elliptical shape, and with good interlayer and intralayer bonding observed. Only small voids between the beads were observed. This was the consequence of the printing orientation (i.e., flatwise) that is not affected by overhanging effects, and therefore is not influenced by the shear thinning behavior of the polymers. The white and black filaments resulted in better mechanical properties compared with the green filaments. ANOVA analysis showed that the white and black filaments displayed average mechanical properties that were not significantly different for both the tensile modulus (*p*-value = 0.106) and the ultimate tensile strength (*p*-value = 0.615). The specimens made by the green filament displayed significantly lower strength than the other specimens tested (Figures S1 and S2). The differences in the mechanical properties can be explained as the results of the different structure of the PLA used for the green filaments, and of the absence of the mineral filler, which can act as reinforcements for the printed parts.

**Figure 10:**
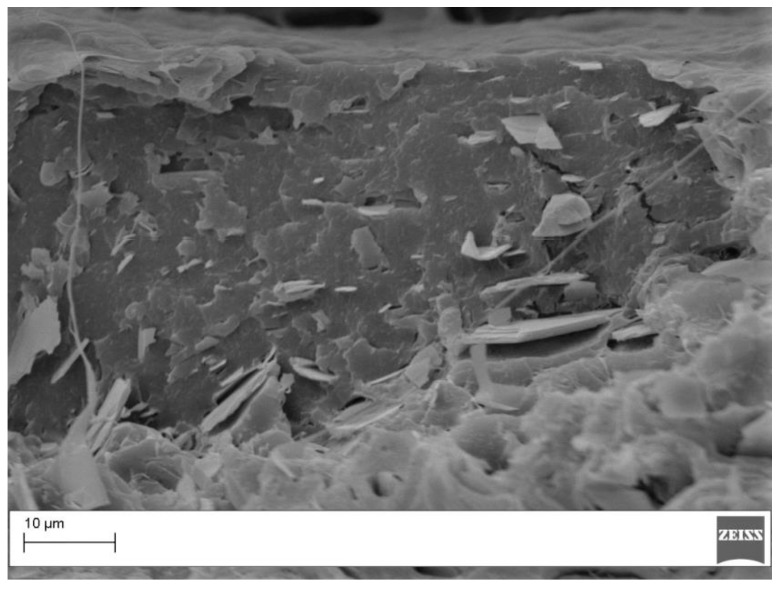
Tensile fractured surface for the black specimens (magnification 3000×).

Figure 10 shows the SEM analysis of the tensile fractured surface for the black specimens. The presence of talc platelet bonded to the matrix is evident, and confirmed the findings on the formulations. The picture clearly showed that the talc platelet was aligned alongside the extrusion direction due to the shear-induced alignment during the extrusion in the nozzle [32]. The talc platelets showed a clean surface and some pull-out. This is the consequence of the use of unfunctionalized talc.

## 4. Conclusions

Three different commercial filaments for FDM were tested and fully characterized. The printing test showed good performances for two filaments (white and black ones), while one (green) failed to print good quality specimens. The filaments were thoroughly characterized by rheology, showing a marked difference between the specimens. The best printing filaments showed a marked shear thinning behavior that was correlated to the presence of mineral fillers in the formulation, as confirmed by TGA and SEM analysis. The high viscosity in the low-frequency range seems to avoid the defects in the overhang printing zones that were observed for the unfilled filaments. The fillers dispersed in the filaments also improved the tensile properties of the printed specimens. Also, DTA of the green sample showed a completely different calorimetric behavior: there was a dramatic decrease in the melting temperature, as well as an increase of the *T*_g_ value with respect to the other two samples, which could be probably due to the absence of talc in the polymer formulation. The findings of this paper support the conclusion that unmodified polymer filaments are not the best choice for FDM. The research should investigate further the optimization of the formulations to exploit the best performances of plastics used for FDM.

## Figures and Tables

**Table 1:** Printing conditions for the preparation of the specimens.

Specimen	Speed [mm/s]	In Fill [%]	In Fill Type	Raster Angle [deg]	Nozzle T [°C]	Bed T [°C]	Layer Height [µm]
Yoda	35–45	10	Grid		210	50	120
Tensile tests	35–45	100		0/90	210	50	120

**Table 2:** Glass transition temperature (*T*_g_), crystallization temperature (*T*_c_), melting temperature (*T*_m_), temperature at 5% mass loss (*T*_5%_), and residue % at 600 °C of the investigated samples.

Filament	*T*_g_ [°C]	*T*_c_ [°C]	*T*_m_ [°C]	*T*_5%_ [°C]	Residue [%]
**White**	58.6	86.2	170.0	334.3	10.7
**Black**	59.9	85.6	168.6	311.7	10.8
**Green**	63.9	106.8	148.2	303.5	0

**Table 3:** Ultimate tensile strength and Young modulus for the printed samples.

Filament	Ultimate Tensile Strength [MPa]	Young Modulus [GPa]
**White**	32.12 ± 3.80	3.40 ± 0.14
**Black**	34.43 ± 3.38	3.73 ± 0.14
**Green**	28.37 ± 1.46	2.66 ± 0.11

## Supplementary Materials

The following are available online at http://www.mdpi.com/1996-1944/11/7/1191/s1, Figure S1: Tukey Simultaneous 95% Cls Difference of Means for E, Figure S2: Tukey Simultaneous 95% Cls Difference of Means for UTS.
