# Supplementary material for: Polylactide (PLA) Filaments a Biobased Solution for Additive Manufacturing: Correlating Rheology and Thermomechanical Properties with Printing Quality
Source: Materials (Basel). 2018 Jul 11;11(7):1191. doi: 10.3390/ma11071191 (PMC6073186; doi:10.3390/ma11071191)
Supplement: Supplementary file 1 [file materials-11-01191-s001.pdf]

Supplementary materials

Appendix A

Estimation of the true shear rate at the wall nozzle during FDM

The nozzle can be assumed as a capillary with length L_n= 1.35 mm and diameter D_n=0.4 mm. The apparent shear rate at the wall ($\dot{\gamma}_w^{app}$), i.e., the shear rate in case of Newtonian fluids, can be expressed as:

$$\dot{\gamma}_w^{app} = \frac{32Q}{\pi D_n^3} \tag{1}$$

Q is the volume flow rate, which can be written in terms of filament diameter (D_f) and feeding velocity (v_f) as:

$$Q = \frac{\pi D_f^2}{4} v_f \tag{2}$$

Substituting Eq. A.2 into Eq. A.1, and assuming D_f=2 mm (check) and v_f=40 mm/s, we get $\dot{\gamma}_w^{app} \sim 2 \times 10^4$ 1/s. This value needs to be corrected to account for the non-Newtonian feature of the fluid. For PLA, a power-law behavior ($\eta \sim \dot{\gamma}^{n-1}$, n being the flow index) can be assumed [20]. In such cases, the Mooney-Rabinowitsch correction provides the following simple expression for the true shear rate:

$$\dot{\gamma}_w^{true} = \frac{3n + 1}{4n} \dot{\gamma}_w^{app} \tag{3}$$

Substituting n=0.25 as found through capillary rheometer experiments, the shear rate at the nozzle wall is $\dot{\gamma}_w^{true} \sim 3.6 \times 10^s$ 1/s.

Appendix B

Statistical analysis

DOE analyis was set on the categorical factor filament type as reported below

Categorical Factor: Filament	
**Level 1**	White
**Level 2**	Black
**Level 3**	Green

The responses were the ultimate tensile strength and the Young modulus (UTS)

Responses		
**E**	Ultimate Tensile Strength	[MPa]
**UTS**	Young Modulus	[GPa]

The residual analysis for the response E is reported below

Residual analysis for Response E

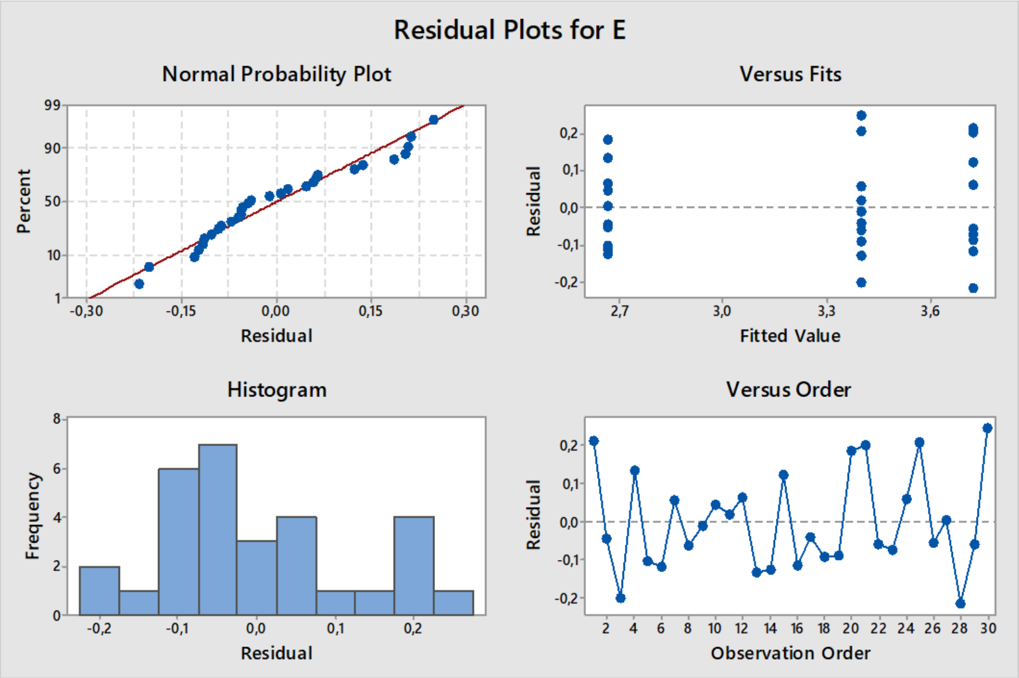

The graphs show that: the error distribution is symmetric and that only small deviations from normal distribution are observed. The graph of residual versu observation order show no residual correlation. The variance is homogenous

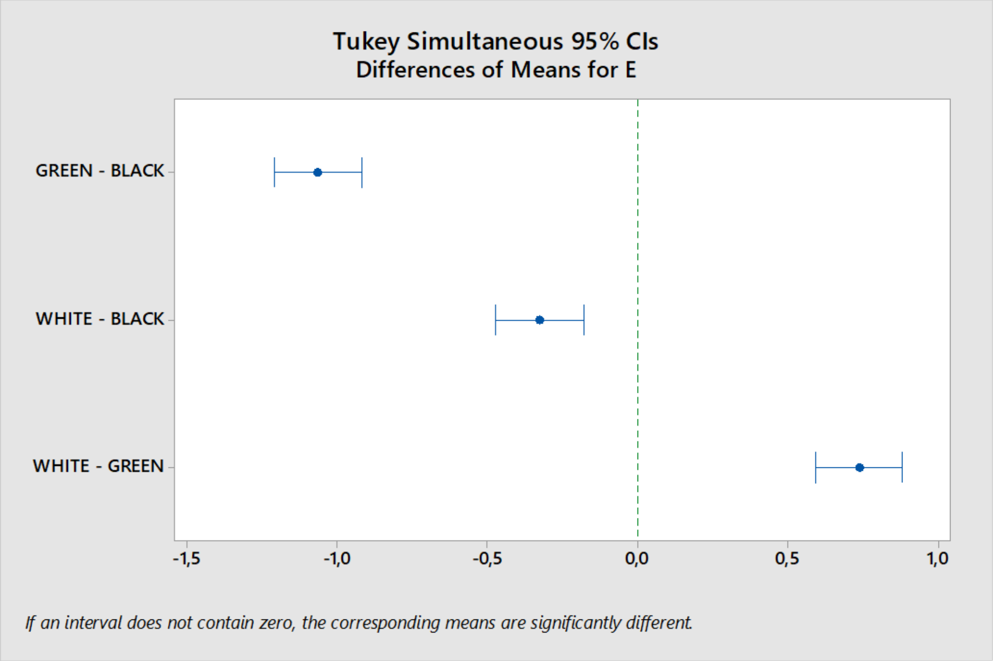

Figure S1. Tukey Simultaneous 95% Cls Difference of Means for E.

The residual analysis for the response UTS is reported below

Residual analysis for Response UTS

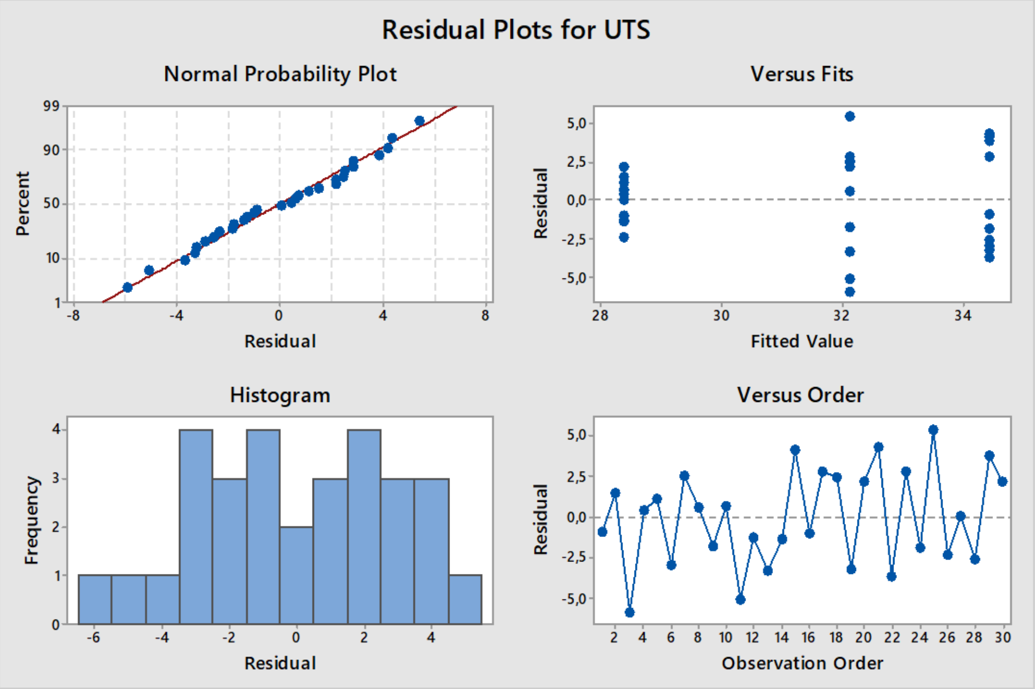

The graphs show that: the error distribution is symmetric and that only small deviations from normal distribution are observed. The graph of residual versu observation order show no residual correlation. The variance is homogenous

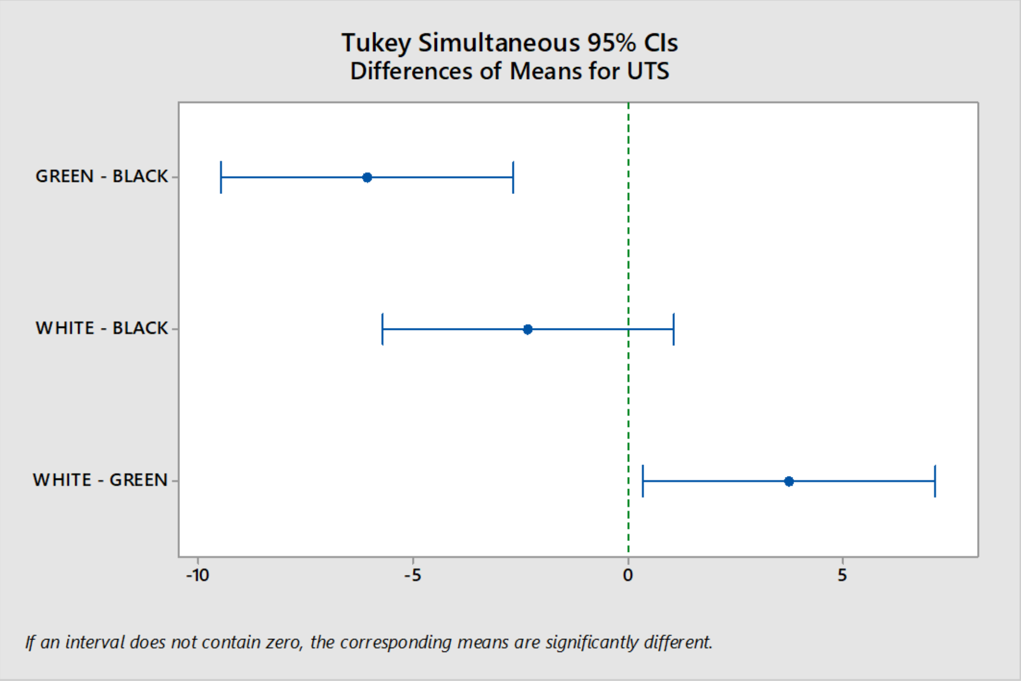

Figure S2. Tukey Simultaneous 95% Cls Difference of Means for UTS.
